# Spatial and spectral characteristics in realizations of broadband terahertz spectroscopy on a subwavelength scale

**DOI:** 10.1038/s41598-023-39396-9

**Published:** 2023-07-30

**Authors:** Alexis N. Guidi, Michael E. Mitchell, Jonathan F. Holzman

**Affiliations:** Integrated Optics Laboratory, School of Engineering, The University of British Columbia, Kelowna, BC V1V 1V7 Canada

**Keywords:** Terahertz optics, Biophotonics

## Abstract

In this work, we take aim at the fundamental challenge for realizations of broadband terahertz (THz) spectroscopy on a subwavelength scale. We introduce apertured THz microjets in this effort to resolve the fundamental limits of spatial resolution and spectral bandwidth. The THz microjets are formed as intense foci at the rear of engineered (microcomposite) spheres and are coupled through subwavelength (circular) apertures. Such coupling enables effective transmission of THz power through samples with broad spectral bandwidths and fine spatial resolutions. We show that the apertures function as high-pass filters, with their diameter *d* enabling strong transmission above a cutoff frequency *f*_c_. Our theoretical and experimental results reveal that the values for *d* and *f*_c_ are prescribed by a fixed spatial-spectral product *df*_c_, whereby reductions in *d* (to improve the spatial resolution) can raise *f*_c_ into the targeted spectrum (at the expense of spectral bandwidth). We use this understanding to demonstrate broadband (0.3–0.7 THz) THz spectroscopy of lactose at the subwavelength (365 µm) scale. These results for apertured THz microjets represent a 20-fold improvement in spatial resolution over analogous apertured THz plane waves. Overall, our findings show promise for studies of carcinogenesis, pathogenesis, and the like.

## Introduction

Terahertz (THz) radiation is a highly effective probe for biomolecular spectroscopy^1–5^. Its frequencies, spanning 0.3–3 THz, couple to many of the key rotational and vibrational modes of biomolecules^3,6–8^, while its photon energies, from 1.24 to 12.4 meV, lay well below the typical ionization energies of cells^3,5,8^. This yields great potential in characterizations of simple biomolecular compounds, such as our own studies of glucose^9^, to far more complex tissues, showing physiological markers of disease^2,7,10–12^. Unfortunately, the relatively low frequencies of the THz spectrum, spanning 100–1000 µm, yield far longer wavelengths than the visible and infrared spectra. This makes it difficult to focus THz radiation down to the scale of cells^10,13^, and even more difficult to implement broadband THz spectroscopy on this subwavelength scale.

There is an ongoing pursuit to overcome this challenge of implementing THz spectroscopy on the subwavelength scale. The pursuits often make use of near-field THz imaging with various forms of tips and probes, and they have been successful in achieving fine spatial resolutions. However, such pursuits also show a pressing challenge in enabling efficient coupling of THz power from the (macroscopic) scale of the incident beam down to the (microscopic) scale of the sample/probe. For example, von Ribbeck et al.^14^ implement scattering-type scanning near-field optical microscopy (SNOM) with a nanotip to achieve nanoscale resolutions, but the results suffer from a lack of THz power. The authors refer to this as insufficient signal strength for “true sample scanning” (p. 3437). Similarly, Mitrofanov et al.^15^ implement SNOM with an aperture in a metallic screen, but the screen reflects most of the incident THz power. The authors acknowledge this challenge and attribute it to extremely weak coupling between the THz radiation and the aperture. Similar studies achieve microscale^16^ and nanoscale^17^ spatial resolutions, but these studies also report low THz powers. Overall, there is a trend in the literature whereby near-field THz imaging can offer fine spatial resolutions, but only at the expense of THz power (and signal strength). Our work confronts this challenge with intense THz microjets, which can be coupled to apertures or engineered near-field tips and probes to drive appreciable THz power down to the subwavelength scale. (There is potential for our work to be applied to emerging concepts in nonlinear ghost imaging^18–20^, but the utility would mainly be in allowing for diffraction-limited imaging with an increased numerical aperture^19^).

In this work, we recognize the above challenge and investigate a potential solution to realize broadband THz spectroscopy on the subwavelength scale. We do so by focusing THz radiation through engineered dielectric spheres, resulting in highly intense THz microjets, which are then coupled through apertures to bring about further constriction and finer spatial resolution. We analyse the spatial and spectral characteristics of THz microjets and apertures used in isolation and coupled together to glean details on the fundamental limits of spatial resolution and spectral bandwidth. This is done by characterizing Kramers–Kronig (K–K) consistency of refractive and absorptive spectra for varied levels of aperturing. The results reveal that the fine spatial resolutions and broad spectral bandwidths that are sought for THz spectroscopy have a complex interplay—and must be considered together. Such findings are used in this work to optimize the spatial and spectral characteristics of the coupled THz microjets and apertures, and then demonstrate broadband (0.3–0.7 THz) THz spectroscopy of lactose on a subwavelength (365 µm) scale.

## Methodology

The THz spatial and spectral characteristics in this work are acquired from a THz time-domain spectroscopy (THz-TDS) system that is seeded by a mode-locked titanium sapphire laser (Mai Tai HP, Spectra-Physics Inc.) operating with a central wavelength of 780 nm, a repetition rate of 80 MHz, and 70-fs-duration laser pulses. The THz-TDS components and their functionalities are detailed in our prior study^21^. In this work, we analyse refraction and absorption characteristics over the lowest frequencies of the THz spectrum, 0.3–0.7 THz. These frequencies yield the longest wavelengths and are thus the most challenging to resolve at fine spatial resolutions. We carry out these spectral analyses on samples of lactose powder, given that this biomolecular compound has distinguishing features in our targeted spectrum of 0.3–0.7 THz.

The transmission characteristics of the samples are extracted from the THz-TDS system and then used to quantify the sample’s refractive index *n*(*f*) and extinction coefficient *κ*(*f*) as a function of frequency *f*. To this end, time-domain waveforms of the electric field are recorded with and without the sample being present, and the results are Fourier transformed into the sample spectrum *E*_s_(*f*) and reference spectrum *E*_r_(*f*), respectively. These complex spectra are then used to define the complex refractive index of the sample *ñ*(*f*) = *n*(*f*)–j*κ*(*f*). Here, the real component is the refractive index^22,23^

1a $$n(f) = 1 - \frac{{\angle E_{{\text{s}}} (f) - \angle E_{{\text{r}}} (f)}}{{k_{0} \ell }}$$

and the imaginary component is the extinction coefficient

1b $$\kappa (f) = \frac{ - 1}{{k_{0} \ell }}\ln \left( {\frac{{(1 + n(f)^{2} }}{4n(f)}\,\frac{{|E_{{\text{s}}} (f)|}}{{|E_{{\text{r}}} (f)|}}} \right),$$

where *k*_0_ = 2π*f*/*c* is the magnitude of the wavevector in free space, given *c* as the speed of light in free space, and *ℓ* is the sample thickness. The operators | · | and ∠(·) denote the magnitude and phase of their complex arguments, respectively. We characterize the material here by the extinction coefficient *κ*(*f*), as it is the loss-dependent counterpart to the phase-dependent refractive index *n*(*f*). Nonetheless, the extinction coefficient *κ*(*f*) can be readily transformed to the often-used (power) absorption coefficient *α*(*f*) = 2*k*_0_*κ*(*f*). The details and derivations of Eqs. (1a) and (1b) are shown in Supplementary Discussion S1.

It is important to note that the spectra for refractive index *n*(*f*) and extinction coefficient *κ*(*f*) are indelibly linked, i.e., complete knowledge of one fully defines the other. This link is made by way of relations from Kramers and Kronig, as detailed by Lucarini et al.^24^ and Dressel et al.^25^. These K–K relations define the refractive index from knowledge of the extinction coefficient, via

2a $$n(f) = 1 + \frac{2}{\pi }{\text{P}}\int\limits_{{f_{{\text{c}}} }}^{\infty } {\frac{f^{\prime}\kappa (f^{\prime})}{{f^{{\prime}{2}} - f^{2} }}df^{\prime}} ,$$

and the extinction coefficient from knowledge of the refractive index, via

2b $$\kappa (f) = \frac{ - 2f}{\pi }{\text{P}}\int\limits_{{f_{{\text{c}}} }}^{\infty } {\frac{n(f^{\prime}) - 1}{{f^{{\prime}{2}} - f^{2} }}df^{\prime}} ,$$

where P∫(·)d*f*' denotes integration for the Cauchy principal value, *f* is the frequency in the sought spectrum, and *f*' is the frequency in the known spectrum. Of note to this work is the cutoff frequency *f*_c_ in the lower integral limits of Eqs. (2a) and (2b). This cutoff frequency comes about from our use of aperturing and its manifestation as a high-pass filter, passing electromagnetic waves with frequencies above *f*_c_. With such definitions, we measure the refractive index *n*(*f*) and extinction coefficient *κ*(*f*) for a given aperture diameter *d*, and then use Eqs. (2a) and (2b) to test the K–K consistency (and thus the accuracy) of the results^26–28^. Potential artefacts from the cutoff frequency *f*_c_ are then explored. We do not see such diameter-dependent artefacts in the upper integral limits, and so they are simply displayed as infinity. The details and derivations of Eqs. (2a) and (2b) follow the work of Lucarini et al.^26^.

The cutoff frequency considered in this work is modelled according to electromagnetic wave propagation through a circular waveguide of diameter *d* bounded by a perfect conductor. Such a system passes electromagnetic waves with frequencies above the cutoff frequency, *f* > *f*_c_, and attenuates electromagnetic waves with frequencies below the cutoff frequency, *f* < *f*_c_. The attenuation is dominated by the lowest-order transverse-electric (TE) mode, TE_11_, which has a cutoff frequency of

3a $$f_{{\text{c}}} \approx \frac{1.841c}{{\pi dn_{0} }}$$

and an associated spatial-spectral product of

3b $$df_{{\text{c}}} \approx \frac{1.841}{{\pi n_{0} }}c,$$

where the factor of 1.841 is the (approximate) least zero of the derivative of the Bessel function of the first kind and first order, and *n*_0_ is the average refractive index of the sample across the targeted spectrum. (The average refractive index of the sample appears here because it is in contact with the aperture while the aperture's thickness is much less than the wavelength.) The cutoff frequency in Eq. (3a) follows from prior work on THz spectroscopy through apertures^29–31^, while the spatial-spectral product in Eq. (3b) is introduced in this work as a fundamental limit in the pursuit of THz spectroscopy through apertures. This limit can only be reached given due attention to (and a sufficiently high value of) the incident THz beam intensity. Moreover, when this limit is reached, its product shows the need to balance pursuits of fine spatial resolutions (via small *d*) and broad spectral bandwidths (via low *f*_c_). The details and derivations of Eqs. (3a) and (3b) follow the work of Pozar et al.^32^, Das et al.^33^, and Marcuvitz et al.^34^ and are shown in Supplementary Discussion S2.

For this study, the refractive index *n*(*f*) and extinction coefficient *κ*(*f*) are measured for samples with a variety of apertures. The results for each sample are then tested for K–K consistency, via Eqs. (2a) and (2b), while monitoring for potential effects from the aperture and its cutoff frequency, as defined in Eqs. (3a) and (3b). The samples are prepared from α-lactose monohydrate powder (Sigma-Aldrich L2643) that was pressed into 1.3-mm-thick flat discs (with a diameter of 15 mm); the apertures are formed by laser micro-milling holes into 16-µm-thick aluminum film with varied diameters (at a tolerance of ± 4 µm). This film thickness is chosen because it is far larger than the skin depth of the applied THz radiation^35^. The prepared samples and apertures are then tested in two distinct implementations.

**Figure 1:**
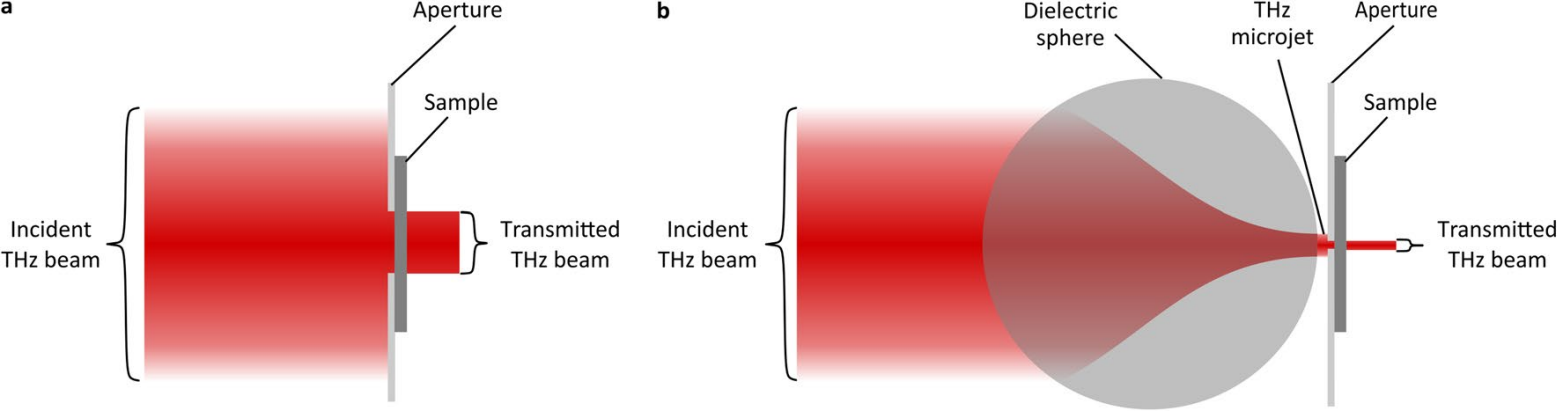
The two implementations for THz-TDS characterizations via aperturing and focusing of THz radiation (in red). The implementation with THz plane waves, shown in (**a**), is a benchmark of comparison as it uses only an aperture to constrict and transmit the THz beam through the sample. The implementation with THz microjets, shown in (**b**), couples a THz microjet through the aperture to further constrict and better transmit the THz beam through the sample.

Figure 1a shows the first implementation. It is a benchmark of comparison for this study, in that it has simple THz plane waves incident upon apertures of varied diameters. The THz plane waves are formed from a collimated THz beam with a full-width-at-half-maximum (FWHM) of 25.4 mm, while the apertures have diameters of *d* = 13.4, 10.0, 8.5, 7.0, 5.0, 4.0, and 3.0 mm. Figure 1b shows the second implementation. It is a test of coupled THz microjets and apertures—in that it has THz microjets focused through apertures of varied diameters. These THz microjets take the form of intense foci at the rear of engineered dielectric (microcomposite) spheres. Following our prior work^36,37^, the 20-mm-diameter spheres are fabricated out of microcomposite material having SiO_2_ microparticles embedded in an ultra-high-molecular-weight polyethylene host. The microcomposite and its refractive index are optimized to yield intense focusing of the THz radiation with a small focal spot size (480 µm at 0.5 THz) and broad bandwidth (0.3–0.7 THz). The THz microjets are then coupled through apertures having diameters of *d* = 1780, 715, 510, 365, 260, 186, and 132 µm.

It is worthwhile at this point to comment briefly upon our THz spectroscopy studies in general and the differing abilities of their focusing elements, including parabolic mirrors, high-resolution polymethylpentene (TPX) lenses, and the abovementioned dielectric spheres (forming THz microjets). We have characterized the beam profiles through the focal spots of these elements, via knife-edge scans^38,39^, to extract their focal spot sizes in terms of FWHM values. At a frequency of 0.5 THz, we found that the parabolic mirror, TPX lens, and dielectric sphere had FWHM values of 2,600, 650, and 480 µm, respectively, and that the dielectric sphere was the only focusing element capable of subwavelength focusing over our entire targeted spectrum. Further details of these analyses are shown in Supplementary Discussion S3. In comparing these focusing elements, we define an *intensity gain* as the ratio of peak intensity in the focal spot to incident intensity. In the absence of loss, this intensity gain is roughly the squared ratio of the element's diameter to the element's focal spot size. Our work uses this strategy with (apertured) THz microjets compared to (apertured) THz plane waves, as it gives us a normalization/benchmark of comparison and lets us compare different focusing elements. Ultimately, the THz plane waves should be seen only as a normalization/benchmark, rather than a strategy for THz spectroscopy with a fine spatial resolution.

## Results and discussion

Results are presented within the following two subsections for apertured THz plane waves, as a benchmark of comparison, and apertured THz microjets, in an attempt to resolve spatial and spectral limits.

### Apertured terahertz plane waves

**Figure 2:**
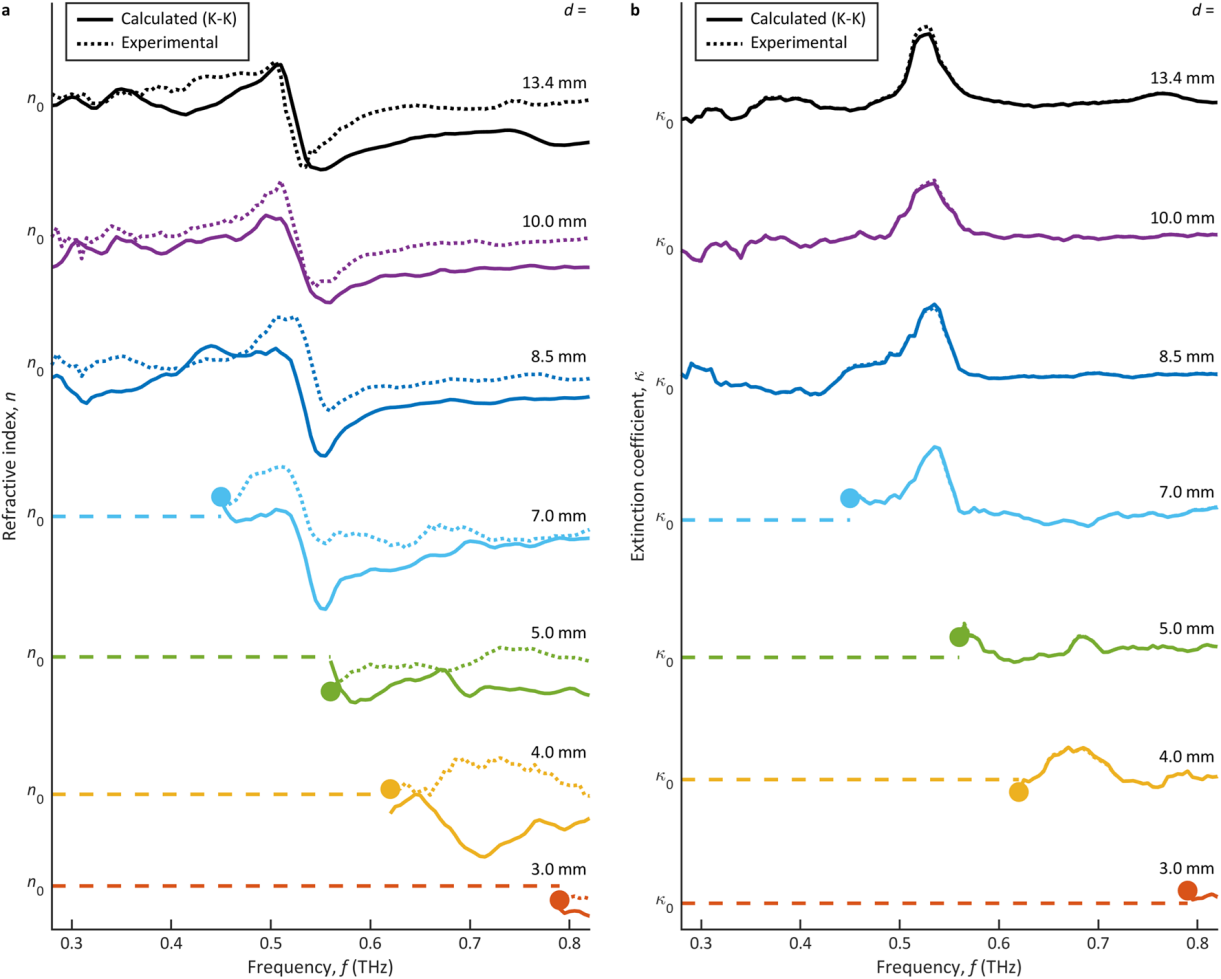
The THz-TDS characterizations of lactose via THz plane waves. The refractive index *n*(*f*) and extinction coefficient *κ*(*f*) are shown in (**a**) and (**b**), respectively, as a function of frequency *f*. The results are presented for aperture diameters of *d* = 13.4, 10.0, 8.5, 7.0, 5.0, 4.0, and 3.0 mm (from top to bottom). Estimates of the cutoff frequency *f*_c_ are shown as solid circles, below which there are spurious results (dashed lines) and above which there are experimental results (dotted lines) and calculated results via the K–K relations (solid lines). The curves are plotted with respect to (and displaced vertically by) *n*_0_ ≈ 1.626 in (**a**) and *κ*_0_ ≈ 0 in (**b**).

Figure 2 shows THz-TDS results for lactose as measured by apertured THz plane waves (with a pre-aperture FWHM of 25.4 mm). Figures 2a and 2b show the refractive index *n*(*f*) and extinction coefficient *κ*(*f*), respectively, as a function of frequency *f* given aperture diameters of *d* = 13.4, 10.0, 8.5, 7.0, 5.0, 4.0, and 3.0 mm. The results for these diameters are shown as solid curves that are plotted with respect to (and displaced vertically by) the average refractive index, *n*_0_ ≈ 1.626, or the average extinction coefficient, *κ*_0_ ≈ 0. Estimates of the cutoff frequency *f*_c_ were obtained by characterizing deviations between the refractive index results for each diameter and the results of the largest aperture diameter, *d* = 13.4 mm. These cutoff frequencies are shown as solid circles in the figure, below which there exists spurious results (at greater than 3% deviation) and above which there exists accurate results (at less than 3% deviation).

Some overall trends are apparent in Fig. 2. The results for the refractive index *n*(*f*) and extinction coefficient *κ*(*f*) are the most accurate at the largest aperture diameter, *d* = 13.4 mm (black). These results show a prominent inflection in *n*(*f*) and an associated peak in *κ*(*f*) at *f* = 0.53 THz. This agrees with the K–K relations^24,40^ of Eqs. (2a) and (2b) and prior observations^41–43^, which attribute these characteristics to a sharp vibrational transition in the lactose crystal. At the same time, we see the noise grow with respect to the signal strength as the frequency decreases, i.e., in moving left across the figure. This is a manifestation of frequency-dependent transmission through the apertures, whereby decreasing frequencies (and increasing wavelengths) exhibit reduced transmission through (and weaker THz powers beyond) the apertures. The growing noise at lower frequencies becomes increasingly obvious as the aperture diameter reduces to *d* = 10.0 mm and then *d* = 8.5 mm. When the aperture diameter drops to *d* = 7.0 mm, we then see the cutoff frequency emerge in the targeted spectrum, at *f*_c_ = 0.450 THz. Further reductions to *d* = 5.0, 4.0, and 3.0 mm raise the cutoff frequency up to *f*_c_ = 0.560, 0.620, and 0.790 THz, respectively, yielding spurious noise over the targeted spectrum. We then conclude that *d* = 7.0 mm defines the smallest achievable spatial resolution for these apertured THz plane waves—as this diameter raises *f*_c_ to the lower edge of the targeted spectrum while smaller diameters drive *f*_c_ into the targeted spectrum. To this end, aperture diameters larger than 7.0 mm successfully transmit the THz beam with no truncation in our targeted spectrum.

The THz-TDS results for lactose as measured by apertured THz plane waves are summarized in Table 1. The cutoff frequency *f*_c_ is displayed for our experimental results from Fig. 2 and for our theoretical results from Eq. (3a). We see that the experimental cutoff frequencies here are significantly higher than those predicted by the theoretical model. We attribute this to the relatively low incident THz beam intensity—which offers little power spectral density near the theoretical cutoff frequencies, *f*_c_ = 0.008 to 0.036 THz. The transmitted THz power at these low frequencies is then below the noise floor in our system. With decreasing aperture diameter, *d*, the theoretical cutoff frequency rises towards our targeted spectrum and the experimental spatial-spectral product descends towards the theoretical spatial-spectral product, *df*_c_ = 0.360*c*. However, the smallest *d* still only gives a theoretical cutoff frequency of *f*_c_ = 0.036 THz and an experimental spatial-spectral product of *df*_c_ = 7.906*c*. Moreover, further reductions in the aperture diameter are not feasible, as smaller diameters transmit less THz power through the aperture and thereby yield less accuracy in the results. This trend is evident from the rightmost column of Table 1, which shows that a reducing aperture diameter yields increasing error off K–K consistency. The values in this column are defined as the root-mean-square percentage error between the experimental refractive index results and the calculated results from the experimental extinction coefficient and the K–K relation in Eq. (2a).

**Table 1:** Spatial and spectral characteristics for apertured THz plane waves.

Aperture diameter, *d* (mm)	Cutoff frequency, *f*_c_ (THz)		Spatial-spectral product, *df*_c_ (× *c*)		K–K error (%)
	Experiment	Theory	Experiment	Theory	
13.4	–	0.008	–	0.360	4.9
10.0	–	0.011	–	0.360	4.0
8.5	–	0.013	–	0.360	4.0
7.0	0.450	0.015	10.507	0.360	4.0
5.0	0.560	0.022	9.340	0.360	7.4
4.0	0.620	0.027	8.272	0.360	7.7
3.0	0.790	0.036	7.906	0.360	7.1

Ultimately, these nominal results show that apertured THz plane waves function in a regime that does not conform to the spatial-spectral product, and significant intensification is needed for the THz beam prior to its aperturing. This can enable further reductions in the aperture diameter without sacrificing the transmitted THz power—and can potentially allow us to reach the limiting spatial-spectral product *df*_c_. The following subsection explores this concept by way of coupled THz microjets and apertures.

### Apertured terahertz microjets

Focusing is the most common approach to intensification of THz beams and it can be readily coupled with aperturing. However, the typical elements for focusing THz radiation, such as polymethylpentene (TPX) lenses, yield focal spot sizes at or above the wavelength scale. This leads to spatial resolutions on the millimetre scale for our targeted spectrum^36,37^. In response to this, we have shown in the past that dielectric spheres can be formed from microcomposite material to enable intense focusing of THz radiation. The focal spots manifest as THz microjets at the rear of the spheres, with sizes on a subwavelength scale^36,37^. Such THz microjets are coupled through apertures of varied diameters within this work.

**Figure 3:**
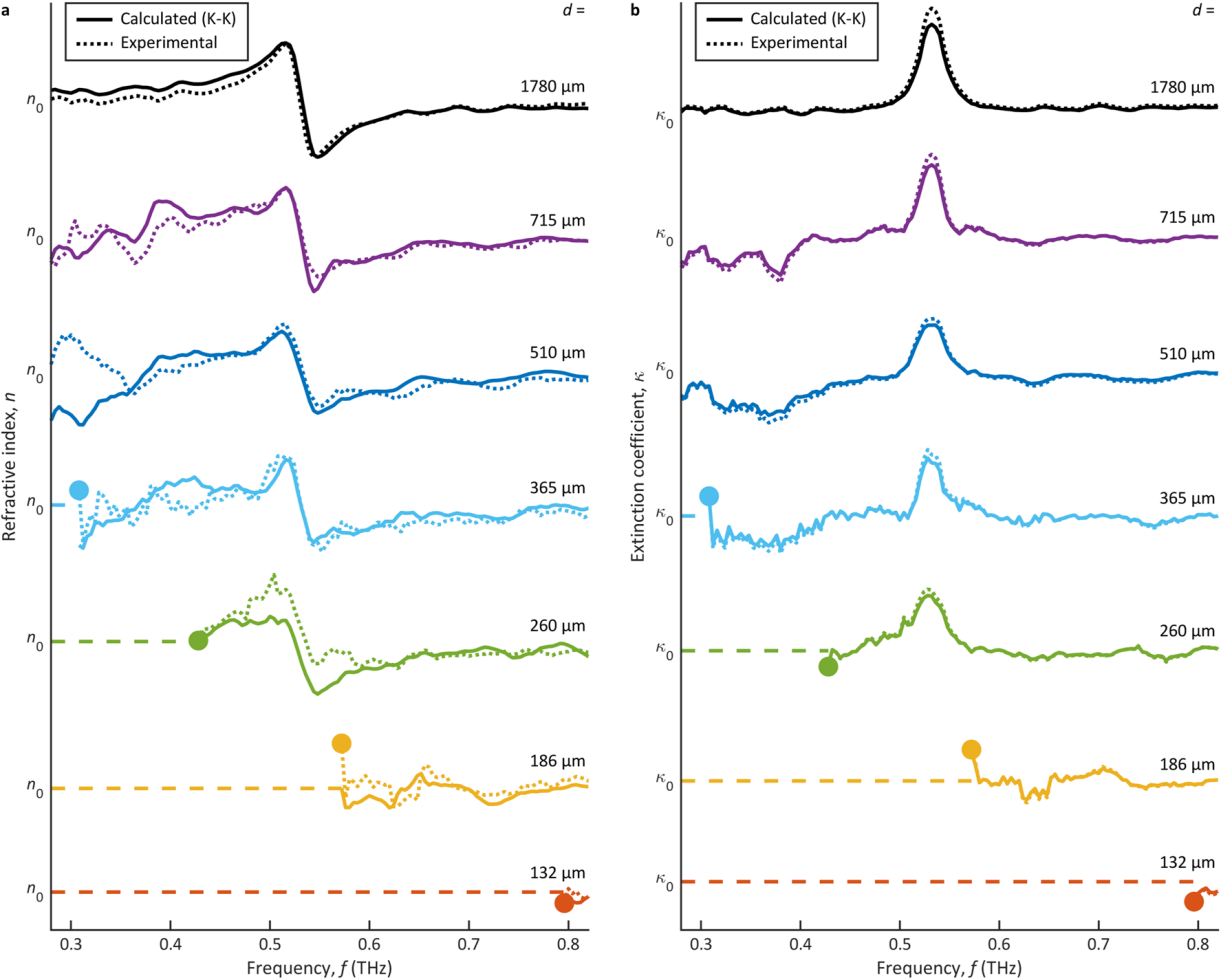
The THz-TDS characterizations of lactose via THz microjets. The refractive index *n*(*f*) and extinction coefficient *κ*(*f*) are shown in (**a**) and (**b**), respectively, as a function of frequency *f*. The results are presented for aperture diameters of *d* = 1780, 715, 510, 365, 260, 186, and 132 µm (from top to bottom). Estimates of the cutoff frequency *f*_c_ are shown as solid circles, below which there are spurious results (dashed lines) and above which there are experimental results (dotted lines) and calculated results via the K–K relations (solid lines). The curves are plotted with respect to (and displaced vertically by) *n*_0_ ≈ 1.532 in (**a**) and *κ*_0_ ≈ 0 in (**b**).

Figure 3 shows THz-TDS results for lactose as measured by apertured THz microjets. Figures 3a and b show the refractive index *n*(*f*) and extinction coefficient *κ*(*f*), respectively, as a function of frequency *f* given aperture diameters of *d* = 1780, 715, 510, 365, 260, 186, and 132 µm. The results for these diameters are shown as solid curves that are plotted with respect to (and displaced vertically by) the average refractive index, *n*_0_ ≈ 1.532, or the average extinction coefficient, *κ*_0_ ≈ 0. Again, estimates of the cutoff frequency *f*_c_ were obtained from a comparison between the refractive index results and those of the largest aperture diameter, *d* = 1780 µm. The cutoff frequencies are shown as solid circles, below which there exists spurious results (at greater than 3% deviation) and above which there exists accurate results (at less than 3% deviation).

The overall trends for the apertured THz microjets in Fig. 3 parallel those of the apertured THz plane waves in Fig. 2. The curves for the largest aperture diameter, *d* = 1780 µm (black), again show a prominent inflection in the refractive index *n*(*f*) and an associated peak in the extinction coefficient *κ*(*f*) at *f* = 0.53 THz. We then see growing noise with respect to the signal strength as the frequency decreases, i.e., in moving left across the figure, and as the aperture diameter decreases to *d* = 715 and 510 µm. When the aperture diameter drops to *d* = 365 µm, we see the cutoff frequency emerge within the targeted spectrum at *f*_c_ = 0.308 THz. Further reductions of the diameter to *d* = 260, 186, and 132 µm then drive the cutoff frequency up to *f*_c_ = 0.428, 0.572, and 0.796 THz, respectively, yielding spurious noise within the targeted spectrum. From such results, we can conclude that *d* = 365 µm defines the smallest achievable spatial resolution for these apertured THz microjets—as this diameter raises *f*_c_ to the lower edge of the targeted spectrum while smaller diameters drive *f*_c_ into the targeted spectrum.

The THz-TDS results for lactose as measured by apertured THz microjets are summarized in Table 2. The cutoff frequency *f*_c_ is displayed for our experimental results from Fig. 3 and for our theoretical results from Eq. (3a). For these apertured THz microjets, unlike the THz plane waves, we see that the experimental cutoff frequencies are relatively close to those predicted by the theoretical model (at a maximum deviation of 0.073 THz). The experimental spatial-spectral products are then close to the theoretical spatial-spectral product, *df*_c_ = 0.383*c*, from Eq. (3b). We attribute this to the smaller aperture diameters that can be used with THz microjets. The THz microjets yield a much higher incident THz beam intensity, which allows us to use significantly smaller aperture diameters while still transmitting a sufficiently high THz power. The accuracy of the results is evident from the rightmost column of Table 2, which shows a relatively low and constant error in the experimental results off K–K consistency. The values in this column are defined in an analogous manner to those detailed in the prior subsection.

**Table 2:** Spatial and spectral characteristics for apertured THz microjets.

Aperture diameter, *d* (µm)	Cutoff frequency, *f*_c_ (THz)		Spatial-spectral product, *df*_c_ (× *c*)		K–K error (%)
	Experiment	Theory	Experiment	Theory	
1780	–	0.064	–	0.383	1.9
715	–	0.160	–	0.383	1.8
510	–	0.225	–	0.383	2.0
365	0.308	0.314	0.375	0.383	1.1
260	0.428	0.441	0.371	0.383	1.4
186	0.572	0.617	0.355	0.383	1.9
132	0.796	0.869	0.351	0.383	1.4

Ultimately, these results show that coupled THz microjets and apertures can enable the intensification and constriction needed for broadband THz spectroscopy on a subwavelength scale. The apertured THz microjets are then characterized by a fundamental limit for spatial resolution and spectral bandwidth—in accordance with the spatial-spectral product *df*_c_.

## Conclusion

In this work, we took aim at the foremost challenge for realizations of broadband THz spectroscopy on the subwavelength scale. To this end, we characterized lactose over a targeted spectrum of 0.3–0.7 THz, as this spectrum exhibits distinguishing features and the greatest challenges in focusing. The characterizations were implemented with apertured THz plane waves, as a benchmark, and apertured THz microjets, to resolve the limits of spatial resolution and spectral bandwidth. Ultimately, our implementations showed a need to consider the levels of both intensification, in keeping the transmitted THz power above the noise floor, and constriction, in balancing desires for fine spatial resolutions and broad spectral bandwidths. This balance manifests through a spatial-spectral product *df*_c_, whereby a reduction in aperture diameter *d* comes at the expense of an increased cutoff frequency *f*_c_.

We found that apertured THz plane waves function in a regime for which reductions in *d* raise *f*_c_, as might be expected, but this occurs for values of the spatial-spectral product *df*_c_ that are high and varied. In our work, the aperture diameter could only be reduced to *d* = 7.0 mm, and the product decreased to *df*_c_ = 10.507*c*, as further reductions raised *f*_c_ well into the targeted spectrum. This truncation of the spectrum sacrificed accuracy in the results, as quantified in our work by K–K consistency. In response to these challenges, we explored intensification by way of coupled THz microjets and apertures.

We found that apertured THz microjets function in a more favourable regime, whereby reductions in *d* raise *f*_c_ in accordance with a low and constant spatial-spectral product *df*_c_. This let us reduce the diameter to *d* = 365 µm and held the product at *df*_c_ = 0.375*c* with less truncation into the targeted spectrum. Essentially, the intensification and constriction realized by the apertured THz microjets enabled the desired broadband (0.3–0.7 THz) THz spectroscopy on a subwavelength (365 µm) scale.

This work shows the implementation of THz spectroscopy as characterizations of refraction and absorption on a subwavelength scale—but such an implementation can be extended to imaging. For example, it is possible to map out the refractive and absorptive characteristics of a sample as a function of space by rastering/translating the sample^44^ with respect to the apertured THz microjet. This would enable integrated THz spectroscopy and imaging with broad bandwidths and fine spatial resolutions. Moreover, it is possible to couple our THz microjets with the near-field tips and probes seen in the literature^14–17,45–49^, as opposed to the simple circular apertures used in this work. Such coupling would enable even finer spatial resolutions without sacrificing THz power (and signal strength). This would be a notable advancement for implementations of THz spectroscopy and imaging. Finally, future studies may also wish to apply apertured THz microjets at higher frequencies and proportionally smaller scales. This can be done by reoptimizing the dielectric spheres for the formation of THz microjets with shorter wavelengths^36^. Such efforts could open the door to highly effective implementations of THz spectroscopy on the cellular scale—where there exist long-standing challenges and great prospects for studies of carcinogenesis^5,10^, pathogenesis^50,51^, and the like.

## Supplementary Information

Supplementary Information.

## Data Availability

The primary data in this study is available within this article and its Supplementary Information. Additional data and details are available from the corresponding author upon reasonable request.
