# Supplementary material for: Spatial and spectral characteristics in realizations of broadband terahertz spectroscopy on a subwavelength scale
Source: Sci Rep. 2023 Jul 30;13:12332. doi: 10.1038/s41598-023-39396-9 (PMC10387480; doi:10.1038/s41598-023-39396-9)
Supplement: Supplementary file 1 — Supplementary Information. [file 41598_2023_39396_MOESM1_ESM.pdf]

scientific reports

# Supplementary Information for

## Spatial and spectral characteristics in realizations of broadband terahertz spectroscopy on a subwavelength scale

Alexis N. Guidi, Michael E. Mitchell, & Jonathan F. Holzman

Corresponding Author: Jonathan F. Holzman

E-mail: jonathan.holzman@ubc.ca

This file includes:

Supplementary Discussion S1

Supplementary Discussion S2

Supplementary Discussion S3

## Supplementary Discussion S1

Details and derivations for the expressions of refractive index $n(f)$ in Eq. (1a) and extinction coefficient $\kappa(f)$ in Eq. (1b) are given in this appendix. In these analyses, we consider the THz radiation to be linearly polarized electromagnetic waves propagating through the THz-TDS system with and without the sample present. For our purposes, we use the Cartesian coordinate system where the electric field is in the $x$-direction. The electric field is recorded at the output of the sample to define the sample waveform as a function of time $t$, which is Fourier transformed to define the sample spectrum $E_s(f)$ as a function of frequency $f$. This is repeated without the sample present to define the reference waveform and its reference spectrum $E_r(f)$.

The sample spectrum is modelled in this work as the product of the incident spectrum on the sample $E_i(f)$, the transmission coefficient for the sample's front interface, the travelling-wave amplitude/phase factor for propagation through the sample, and the transmission coefficient for the sample's rear interface. This gives a sample spectrum of

$$\begin{aligned} E_s(f) &= E_i(f)\left(\frac{2}{1+n(f)}\right)\left(\frac{2n(f)}{1+n(f)}\right)\mathrm{e}^{-\mathrm{j}k_0\tilde{n}(f)\ell} \\ &= E_i(f)\frac{4n(f)}{(1+n(f))^2}\mathrm{e}^{-k_0\kappa(f)\ell}\mathrm{e}^{-\mathrm{j}k_0 n(f)\ell}\ , \end{aligned} \tag{S1.1}$$

where $k_0 = 2\pi f/c$ is the wavevector's magnitude in free space and $c$ is the speed of light in free space, $\tilde{n}(f) = n(f) - \mathrm{j}\kappa(f)$ is the complex refractive index with its constituent refractive index $n(f)$ and extinction coefficient $\kappa(f)$, and $\ell$ is the sample's thickness. The reference spectrum is modelled in this work in a similar manner to the sample spectrum, but with the complex refractive index of the sample and the transmission coefficients set to unity. This gives a simple result for the reference spectrum of

$$E_r(f) = E_i(f)\mathrm{e}^{-\mathrm{j}k_0\ell}\ . \tag{S1.2}$$

To begin defining the material parameters, we take the ratio of the sample and reference spectra, which gives

$$\frac{E_s(f)}{E_r(f)} = \frac{4n(f)}{(1+n(f))^2}\mathrm{e}^{-k_0\kappa(f)\ell}\mathrm{e}^{-\mathrm{j}k_0(n(f)-1)\ell}\ . \tag{S1.3}$$

From this, we see that the difference in phase between the sample and reference spectra defines the refractive index according to Eq. (1a), while the ratio of magnitudes for the sample and reference spectra defines the extinction coefficient according to Eq. (1b).

## Supplementary Discussion S2

Details and derivations for the cutoff frequency $f_c$ in Eq. (3a) and spatial-spectral product $df_c$ in Eq. (3b) are given in this appendix. The derivations consider electromagnetic wave propagation through an aperture, modelled as a circular waveguide of diameter $d$ bounded by a perfect conductor. The fields are defined in terms of the radial dimension $\rho$, azimuthal angle $\phi$, and axial dimension $z$, with propagation in the positive $z$-direction. The lowest order mode to propagate through such a waveguide is a transverse-electric (TE) mode, for which the $z$-direction has an electric field component that is zero, $E_z(\rho,\phi,z) = 0$, and a magnetic field component that is nonzero, $H_z(\rho,\phi,z) \neq 0$. Given these definitions and the linear polarization of the incident THz beam, the propagating wave will be characterized by a superposition of the radial electric field component

$$E_\rho(\rho,\phi,z) = \frac{-\mathrm{j}2\pi f\mu_0}{k_c^2\rho}\frac{\partial H_z(\rho,\phi,z)}{\partial\phi} \tag{S2.1a}$$

and azimuthal electric field component

$$E_\phi(\rho,\phi,z) = \frac{\mathrm{j}2\pi f\mu_0}{k_c^2}\frac{\partial H_z(\rho,\phi,z)}{\partial\rho} \tag{S2.1b}$$

which have the associated radial magnetic field component

$$H_\rho(\rho,\phi,z) = \frac{-\mathrm{j}\beta}{k_c^2}\frac{\partial H_z(\rho,\phi,z)}{\partial\rho} \tag{S2.1c}$$

and azimuthal magnetic field component

$$H_\phi(\rho,\phi,z) = \frac{-\mathrm{j}\beta}{k_c^2\rho}\frac{\partial H_z(\rho,\phi,z)}{\partial\phi}\ . \tag{S2.1d}$$

In these relations, $\mu_0$ is the permeability of free space, $\beta$ is the propagation constant of a given mode, $k_c = (k^2 - \beta^2)^{1/2} = 2\pi f_c n_0/c$ defines the cutoff condition of that mode in terms of the cutoff frequency $f_c$, and $k = 2\pi f n_0/c$ is the magnitude of the wavevector in the waveguide, given the frequency $f$, average refractive index $n_0$, and speed of light in free space $c$. The propagating fields are then fully defined by $H_z(\rho,\phi,z) = R(\rho)F(\phi)\mathrm{e}^{-\mathrm{j}\beta z}$, which evolves according to

$$\nabla^2 H_z(\rho,\phi,z) + k_c^2 H_z(\rho,\phi,z) = 0\ , \tag{S2.2}$$

where $\nabla^2$ is the Laplacian operator.

The solution of (S2.2) can be had via separation of variables, with its dependence on the radial dimension $\rho$ encompassed by $R(\rho)$ and found from

$$\rho^2 \frac{d^2R(\rho)}{d\rho^2} + \rho \frac{dR(\rho)}{d\rho} + R(\rho)(\rho^2 k_c^2 - m^2) = 0 \ . \tag{S2.3a}$$

and its dependence on the azimuthal angle $\phi$ encompassed by $F(\phi)$ and found from

$$\frac{d^2F(\phi)}{d\phi^2} + m^2 F(\phi) = 0 \ . \tag{S2.3b}$$

We then solve (S2.3a) and (S2.3b), and apply knowledge that the solution must be finite at an infinite radius, to obtain

$$H_z(\rho,\phi,z) = J_m(k_c\rho)(A\sin(m\phi) + B\cos(m\phi))e^{-j\beta z} \ , \tag{S2.4}$$

where $A$ and $B$ are constants, and $J_m(\cdot)$ is the Bessel function of the first kind of integer order $m$. We then apply a boundary condition on the conductor by setting the tangential (azimuthal) component of the electric field $E_\phi(\rho,\phi,z)$ to zero at $\rho = d/2$. This condition and (S2.1b) dictate that the derivative of $H_z(\rho,\phi,z)$ along the radial dimension $\rho$ and thus the derivative of the Bessel function of the first kind with respect to its argument, $J'_m(k_c\rho)$, must also be zero at $\rho = d/2$. The least zero to meet this condition has the argument of the Bessel function's derivative, $k_c d/2$, equal to roughly 1.841. This sets a cutoff frequency of $f_c \approx 1.841c/(\pi d n_0)$, in agreement with Eq. (3a), and it sets a spatial-spectral product of $df_c \approx 1.841c/(\pi n_0)$, in agreement with Eq. (3b).

## Supplementary Discussion S3

Through the course of all our studies on THz spectroscopy, we have applied various forms of focusing elements, including parabolic mirrors (Edmund Optics Gold Off-Axis Parabolic Mirror, PFL50.8), high-resolution polymethylpentene (TPX) lenses (BATOP, TPX-D25.4-f10), and our own dielectric spheres (forming THz microjets). We have compiled experimental results for the beam profiles of the three aforementioned elements to quantify their focusing performance. The beam profiles were acquired at the focal spot of each element by way of knife-edge scans, following established procedures[1,2]. The beam profiles and their resulting full-width-at-half-maximum (FWHM) values, from our present study and one of our prior studies[3], are shown in Fig. S1.

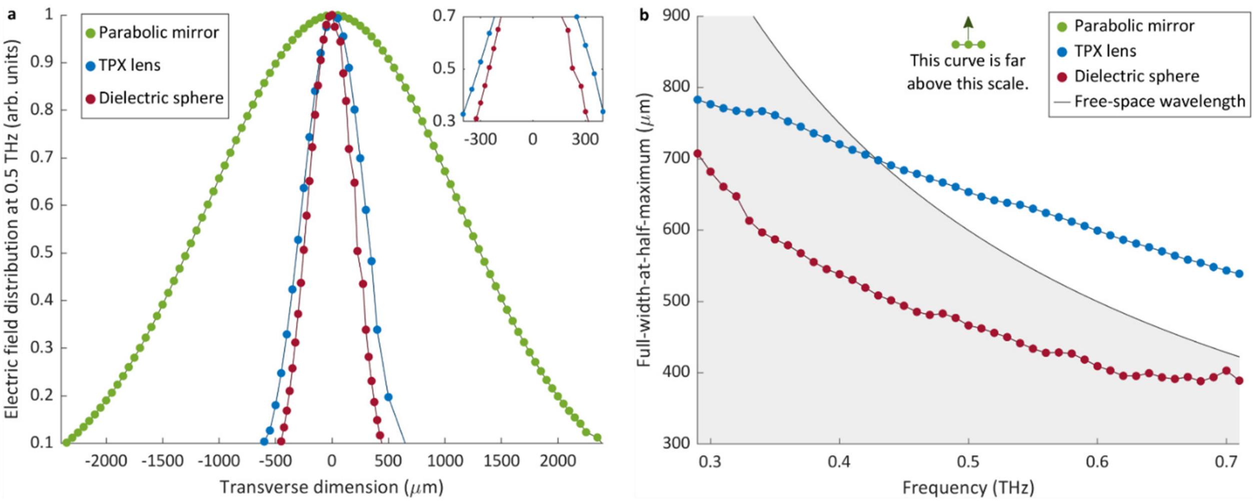


**Figure S1.** The beam profile characterization of various focusing elements measured via knife-edge scans. (**a**) shows the electric field distribution across the THz focal spot as a function of the transverse dimension for the parabolic mirror (green), high-resolution TPX lens (blue), and dielectric sphere (red) at a frequency of 0.5 THz. The inset shows a closeup of the curves for the TPX lens and the dielectric sphere near the FWHM. (**b**) shows the FWHM values of the THz focal spot as a function of frequency for the high-resolution TPX lens (blue) and the dielectric sphere (red). Results are not shown for the parabolic mirror (green), given that its results are well above the desired subwavelength region, which is demarcated by a dark grey curve and shaded in light grey. For both (**a**) and (**b**), the solid circles indicate experimental data points, while the solid curves guide the eye.

Figure S1a shows the electric field distribution across the THz focal spot as a function of the transverse dimension for two common focusing elements, a parabolic mirror (green) and a high-resolution TPX lens (blue), as well as our dielectric sphere (red), at a frequency of 0.5 THz. The solid circles indicate experimental data points, while the solid curves guide the eye. The inset shows a closeup of the curves for the TPX lens and the dielectric sphere near the FWHM. Overall, we see that the parabolic mirror, TPX lens, and dielectric sphere yield FWHM values of 2,600, 650, and 480 μm, respectively. Thus, the dielectric sphere achieves the smallest focal spot and is the only element to achieve subwavelength focusing, given that the wavelength for 0.5 THz is 600 μm.

Figure S1b elaborates on these focusing characteristics by showing the FWHM values of the THz focal spot as a function of frequency for the high-resolution TPX lens (blue) and dielectric sphere (red). Results are not shown for the parabolic mirror (green), given that its results are well above the desired subwavelength region, which is demarcated by a dark grey curve and shaded in light grey. The solid circles indicate experimental data points, while the solid curves guide the eye. The results show that the FWHM values of the TPX lens are only in the subwavelength region for roughly one-third of our targeted spectrum. In contrast, the FWHM values of the dielectric sphere remain in the subwavelength region over the full spectrum.

## References


1. Khosrofian, J. M. & Garetz, B. A. Measurement of a Gaussian laser beam diameter through the direct inversion of knife-edge data. *Appl. Opt.* **22(21),** 3406–3410 (1983).
2. Tofani, S., Zografopoulos, D. C., Missori, M., Fastampa, R., & Beccherelli, R. Terahertz focusing properties of polymeric zone plates characterized by a modified knife-edge technique. *J. Opt. Soc. Am. B* **36(5),** D88–D96 (2019).
3. Mitchell, M. E., Bergen, M. H., Reich, J., & Holzman, J. F. Terahertz microjets as realizations of subwavelength focusing in the terahertz spectrum. *IEEE Trans. Terahertz Sci. Technol.* **13(1),** 50–57 (2023).
